# Icariin Promotes the Osteogenic Action of BMP2 by Activating the cAMP Signaling Pathway

**DOI:** 10.3390/molecules24213875

**Published:** 2019-10-28

**Authors:** Meng Chen, Yazhou Cui, Hui Li, Jing Luan, Xiaoyan Zhou, Jinxiang Han

**Affiliations:** 1 School of Traditional Chinese Medicine, Shandong University of Traditional Chinese Medicine, Jinan 250355, China; cm13579.student@sina.com; 2 Shandong Medicinal Biotechnology Center, Key Laboratory for Biotech-Drugs of the Ministry of Health, Jinan 250062, China; 3 Department of Toxicology, Shandong Center for Disease Control and Prevention, Jinan 250014, China

**Keywords:** Icariin (ICA), Osteoblast differentiation, BMP2, cAMP/PKA/CREB

## Abstract

Icariin (ICA) is the main active flavonoid glucoside from herbs of the genus *Epimedium*; in traditional Chinese medicine, these herbs have long been prescribed for the treatment of bone fractures and osteoporosis. Several studies have shown that treatment with ICA can increase osteogenic differentiation and reduce bone loss in vivo and in vitro. However, the definite signaling pathway of this osteogenic effect remains unclear. In this study, we selected bone morphogenetic protein 2 (BMP2)-induced osteoblastic differentiation of multipotent mesenchymal progenitor C2C12 cells as a model of osteoblast differentiation. We investigated the effects of ICA on C2C12 cells osteogenic differentiation and the underlying molecular mechanisms. We found that ICA could enhance BMP2-mediated osteoblastic differentiation of C2C12 cells in a dose-dependent manner. Treatment with ICA activated the cAMP/PKA/CREB signaling axis in a time-dependent manner. Blocking cAMP signaling using the PKA selective inhibitor H89 significantly inhibited the stimulatory effect of ICA on osteogenesis. Therefore, the osteoinductive potential and the low cost of ICA indicate that it is a promising alternative treatment or promoter for enhancing the therapeutic effects of BMP2.

## 1. Introduction

Bone disorders, especially osteoporosis, are becoming increasingly prevalent as the aging population grows, and bone fractures also occur frequently [1]. Osteoporosis contributes significantly to global healthcare costs, and the treatment of osteoporosis remains a great challenge [2]. Thus, new bone anabolic drugs need to be developed for the prevention and treatment of osteoporosis.

Bone morphogenetic protein 2 (BMP2) is one of the most important growth factors that induce osteoblast differentiation and stimulate bone or cartilage formation [3,4]. BMP2 has been widely used in the clinic to promote bone formation. However, the therapeutic dose of BMP2 is very expensive, and there are some life-threatening side effects that can occur with larger doses [5,6,7]. Hence, it is necessary to find additional agents or methods for the improvement of BMP2 itself to intensify the osteogenic effects of BMP2. The combination of BMP2 and these small molecules can promote BMP2-mediated osteoblast differentiation and reduces the risk of its side effects in the treatment of bone diseases. From a clinical perspective, these small molecules that regulate BMP2 activity provide powerful tools for the treatment of osteoporosis and fracture repair.

**Figure 1:**
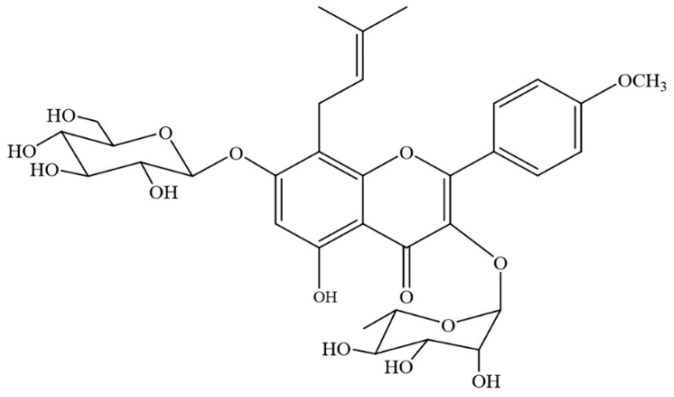
The chemical structure of icariin (ICA).

Icariin (ICA) (the chemical structure is shown in Figure 1) is the main active flavonoid glucoside from herbs of the genus *Epimedium*; these herbs are used in traditional Chinese medicine and have long been prescribed for the treatment of bone fractures and osteoporosis as a bone-protecting agent. In addition to promoting bone regeneration and repair, ICA also has many beneficial pharmacological and biological activities, including neuroprotective effects [8,9]; protective effects against atherosclerosis [10]; antitumor effects [11,12]; anti-inflammatory and antioxidant effects [13,14]; and improved sexual function effects [15]. Several animal and cell studies have shown that treatment with ICA can increase osteogenic differentiation and reduce bone loss in vivo and in vitro [16,17,18]. The extremely low cost of ICA and its strong bone regenerative effects indicate its potential in clinical applications.

The cAMP/PKA/CREB axis is a vital pathway that regulates osteogenic differentiation and mineralization. Several previous studies have reported that PTH affects osteoblastic cells by activating the cAMP/PKA/CREB axis [19,20]. Several studies have reported that pretreatment of human mesenchymal stem cells (MSCs) with cAMP analogs, adenylate cyclase activators or phosphodiesterase inhibitors can enhance bone formation [21,22,23]. 

In the present study, we combined ICA and BMP2 to promote osteogenesis for the first time. We aimed to determine whether ICA could enhance the osteogenic induction of BMP2 in C2C12 cells. We concluded that the combination of ICA and BMP2 could promote BMP2-mediated osteoblast differentiation in a dose-dependent manner. We also found that ICA stimulates BMP2-mediated osteogenesis by activating the cAMP/PKA/CREB signaling pathway. Therefore, ICA is a promising candidate as an alternative for BMP2 or as a promoter for enhancing the therapeutic effects of BMP2. This combination will reduce the risk of side effects from BMP2 in the treatment of bone diseases such as osteoporosis.

## 2. Results

### 2.1. ICA Improves the BMP2-Mediated Osteogenic Differentiation of C2C12 Cells in a Dose-Dependent Manner 

#### 2.1.1. Successful Model of Osteoblast Differentiation with BMP2-Treated C2C12 Cells 

**Figure 2:**
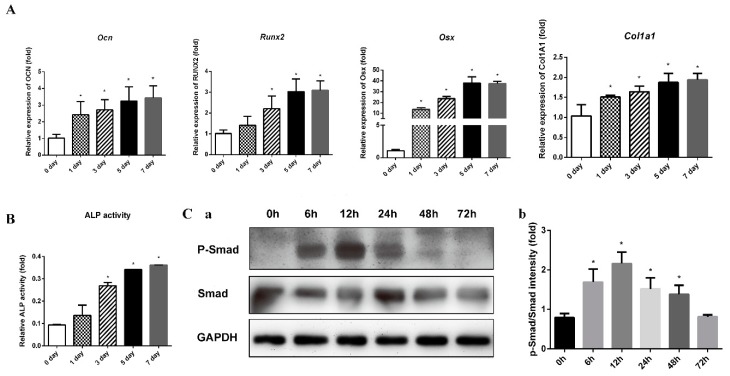
BMP2 induced osteoblastic differentiation of the C2C12 cells. *Ocn*, *Runx2*, *Osx* and *Col1a1* mRNA levels (**A**) and the ALP activity (**B**) at 0, 1, 3, 5 and 7 days of osteogenic induction. (**C-a**) The protein levels of phosphorylated Smad1/5/9 and total Smad1 were evaluated by Western blotting at 0, 6, 12, 24, 48 and 72 h after the addition of BMP2. (**C-b**) Relative density of the protein expression levels. All data are presented as the mean ± SD (*n* = 3). * *p* < 0.05.

BMP2 can block the differentiation of C2C12 myoblasts into mature muscle cells by suppressing the master control genes for myoblast differentiation [24]. The BMP2-mediated differentiation of C2C12 cells into osteoblast-like cells is regarded as a successful model of osteoblast differentiation. As shown in Figure 2A,B, 300 ng/mL of BMP2 significantly induced the representative markers of osteoblast differentiation, such as *Ocn*, *Runx2*, *Osx*, and *Col1a1*, and alkaline phosphatase (ALP) activity. To confirm the stimulatory effect of BMP2-induced osteoblast differentiation, we investigated the related signaling pathways. In the presence of BMP2, cytosolic Smad1/5/9 is phosphorylated, interacts with Smad4, and subsequently translocates into the nucleus, leading to osteoblast differentiation [25]. As shown in Figure 2C, the protein expression levels of phosphorylated Smad1/5/9 and total Smad1 at various times after the addition of BMP2 were evaluated by Western blotting. The results suggested that the activation of the phosphorylated Smad1/5/9-mediated signaling pathway increased after 6 h and peaked after 12 h. However, the protein expression levels of phosphorylated Smad1/5/9 decreased significantly after 24 h. Therefore, in the subsequent experiments, we used the 12 h time point to detect the activation of the Smad signaling pathway.

#### 2.1.2. Cytotoxic Effects of ICA

**Figure 3:**
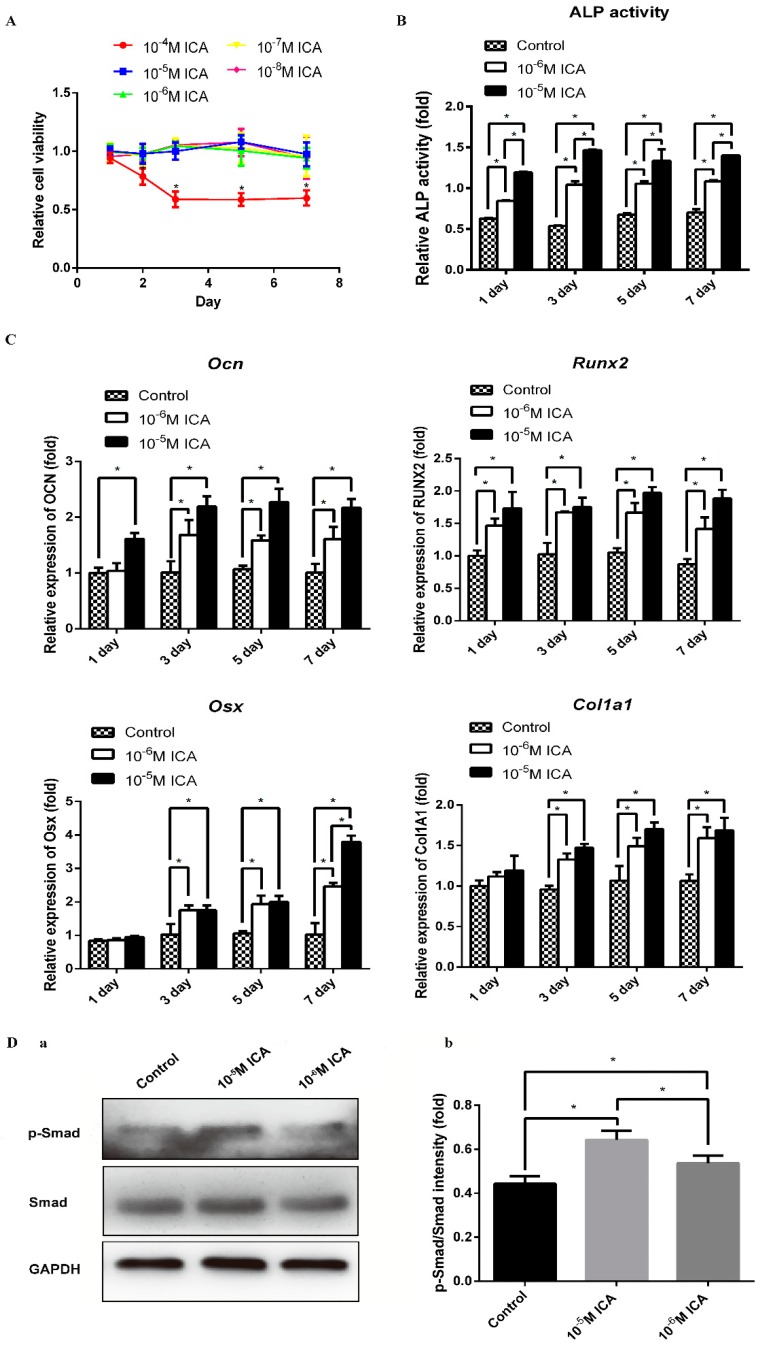
ICA enhances the BMP2-mediated osteogenic differentiation of C2C12 cells. (**A**) Cytotoxic effect of ICA on C2C12 cells. C2C12 cells were cultured in basal medium with various concentrations of ICA (10^−8^, 10^−7^, 10^−6^, 10^−5^ and 10^−4^ M) for 1, 2, 3, 5, and 7 days, then CCK8 assay was performed to test the cytotoxic effect of ICA. The ALP activity (**B**) and the mRNA expression levels (**C**) after 1, 3, 5 and 7 days of osteogenic induction with different concentrations of ICA (0, 10^−6^ and 10^−5^ M). (**D-a**) The protein expression levels of phosphorylated Smad1/5/9 and total Smad1 with different concentrations of ICA (0, 10^−6^ and 10^−5^ M). (**D-b**) Relative density of the protein expression levels. All data are presented as the mean ± SD (*n* = 3). * *p* < 0.05.

Prior to investigating the osteogenic effects of ICA, we examined the cytotoxic effects of ICA on C2C12 cells. We treated cells with different concentrations ranging from 10^−8^ M to 10^−4^ M of ICA for 1, 2, 3, 5 and 7 days. As shown in Figure 3A, the OD values were stable after treatment with 10^−8^ M to 10^−5^ M ICA. However, the OD values decreased when the concentration of ICA was 10^−4^ M. These findings indicated that there is no cytotoxicity when the concentration of ICA was from 10^−8^ M to 10^−5^ M, but the toxic effect of ICA on cell viability were observed at concentrations above 10^−4^ M. The result suggested that the safe concentration range of ICA is lower than 10^−4^ M. Therefore, in the subsequent experiments, we examined the treatment effects of ICA at concentrations of 10^−6^ M and 10^−5^ M ICA on osteoblast differentiation.

#### 2.1.3. ICA Improves BMP2-Mediated Osteogenic Differentiation 

To investigate the promoting effects of ICA on bone formation, we examined the osteogenic differentiation of C2C12 cells immediately after 1, 3, 5, and 7 days of ICA treatment. As shown in Figure 3B,C, among various osteogenic genes, the mRNA expression levels of *Ocn*, *Runx2*, *Osx*, and *Col1a1* increased by the addition of ICA. Consistent with these results, the ALP activity was generally enhanced by ICA treatment. It is worth noting that both the mRNA levels of osteogenic markers and the ALP activity increased in a dose-dependent manner. As shown in Figure 3D, the protein expression levels of phosphorylated Smad1/5/9 were enhanced by ICA treatment in a dose-dependent manner. This result indicated that ICA can stimulate the BMP2-mediated signaling pathways that cooperatively activate osteoblast differentiation. In particular, we found that 10^−5^ M ICA was the optimal concentration for promotion of osteogenic differentiation. Therefore, a concentration of 10^−5^ M ICA was used for the following series of studies.

### 2.2. The Osteogenesis-Stimulating Activity of ICA is Mediated through the cAMP Pathways 

#### 2.2.1. Differentially Expressed Gene (DEG) Analysis by RNA-Seq before and after the Addition of ICA 

**Figure 4:**
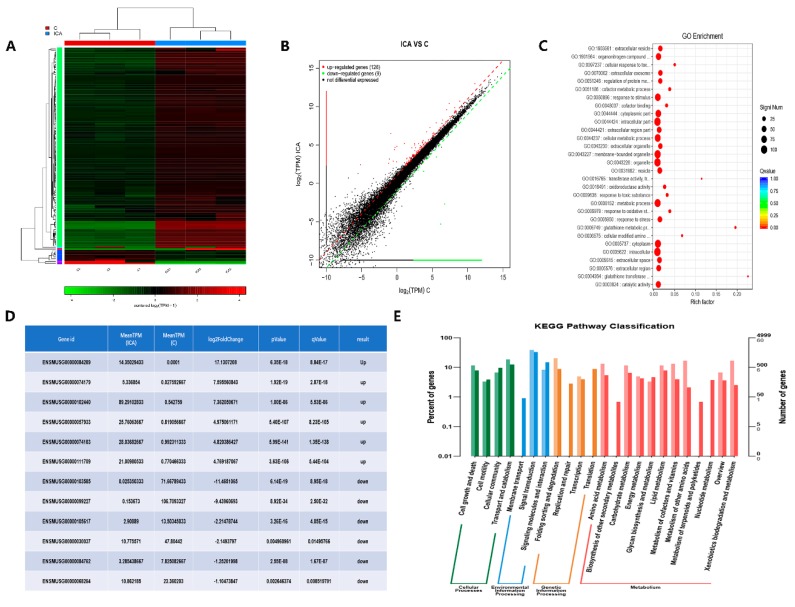
The gene expression profile of ICA-induced osteoblast differentiation was analyzed with RNA-seq technology. (**A**) Heatmap of the differentially expressed genes based on fold changes in the ICA and control group. Red indicates higher expression, and green indicates lower expression. (**B**) Differential expression scatter plot of the comparison groups. A total of 135 genes were differentially expressed after the addition of ICA; 126 were upregulated genes and 9 were downregulated genes. Black represents genes that were not differentially expressed. (**C**) Significant enrichment functional scatter plot. The size of q-value is represented by the color of the dots. The smaller the q-value is, the closer the color is to the red color, and the number of differential genes contained in each function is represented by the size of the dots. (**D**) The 6 most significantly upregulated and downregulated genes. Genes listed from top to bottom according to the significance of the DEGs. (**E**) KEGG pathway classification shows the relationship between metabolic pathways and differentially expressed genes through a classification histogram. Different colors represent different classifications. Light colors represent differentially expressed genes and dark colors represent all genes.

We identified DEGs before and after the addition of ICA with RNA-seq technology to investigate the mechanism underlying the osteoinductive potential of ICA. The results of Figure 4A show that 135 genes were differentially expressed after ICA addition. As shown in Figure 4B, 126 genes were upregulated, and 9 genes were downregulated. According to the GO functional enrichment analysis of the DEGs, we obtained the significantly enriched functional scatter plots, as shown in Figure 4C. We identified the 6 most significantly upregulated and downregulated genes, as shown in Figure 4D. These results could help us to better understand and verify the DEGs. The histogram in Figure 4E shows the relationship between metabolic pathways and DEGs through KEGG pathway classification. We found that the DEGs were enriched in the pathways of cellular processes, environmental information processing, genetic information processing and metabolism after treatment with ICA. Based on the DEG pathway analysis by RNA-seq, we found that many signaling pathways of osteogenic differentiation were upregulated and speculated that the osteoinductive activity of ICA may be associated with the cAMP metabolic pathways.

#### 2.2.2. ICA Activated the cAMP Pathway in Promoting Bone Formation 

**Figure 5:**
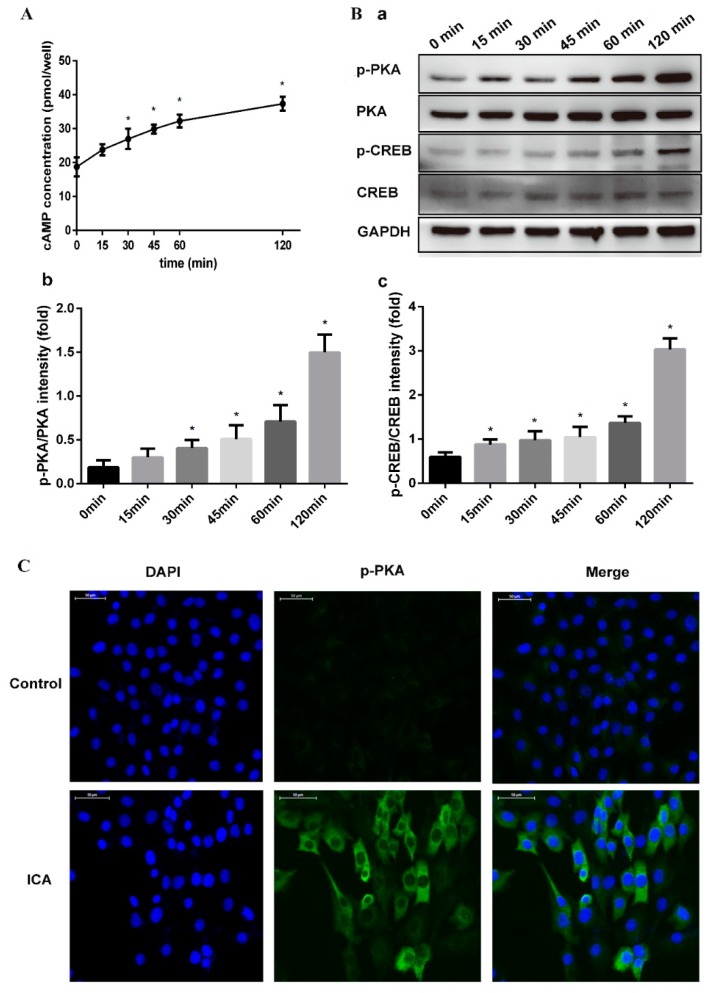
ICA activated the cAMP/PKA/CREB pathway in promoting bone formation. The changes of intracellular cAMP level (**A**) and the protein expression levels of phosphorylated PKA (p-PKA), phosphorylated CREB (p-CREB), total PKA, and CREB (**B-a**) after C2C12 exposure for 0, 15, 30, 45, 60 and 120 min. (**B-b**) Relative density of p-PKA expression levels. (**B-c**) Relative density of p-CREB expression levels. (**C**) The expression of p-PKA after 120 min of ICA treatment by immunofluorescence assays. p-PKA was stained green, and nuclei were stained blue (with DAPI). Bar represents the mean ± SD (*n* = 3). * *p* < 0.05.

We hypothesized that ICA exerts osteogenic effects by enhancing cAMP signaling and that cAMP production was induced by ICA. To confirm this hypothesis, we further examined the activation of cAMP signaling in C2C12 cells simultaneously exposed to ICA (10^−5^ M) and BMP2 (300 ng/mL). First, the changes in the intracellular cAMP levels were analyzed after C2C12 cell exposure at different time periods. As shown in Figure 5A, the cAMP level began to increase after 15 min, and after 30 min, the content was significantly higher than the content at the starting point. The increase continued, reaching an even higher level at 120 min. 

The treatment of C2C12 cells with ICA resulted in significant activation of PKA phosphorylation (p-PKA) and CREB phosphorylation (p-CREB), as shown by the Western blot analysis in Figure 5B. With the duration of the ICA treatment, we found that the expression of p-PKA and p-CREB became more evident, while the expression of total PKA and CREB remained unchanged. Moreover, immunofluorescence assays assessing the expression of p-PKA confirmed this result. As shown in Figure 5C, the expression of the p-PKA was significantly more obvious in the ICA group than in the control group after 120 min. p-PKA did not show visible positive expression in the control. 

### 2.3. PKA Inhibitor (H89) Abrogated ICA-Prompted Osteogenesis via a cAMP/PKA/CREB Signaling Blockade 

**Figure 6:**
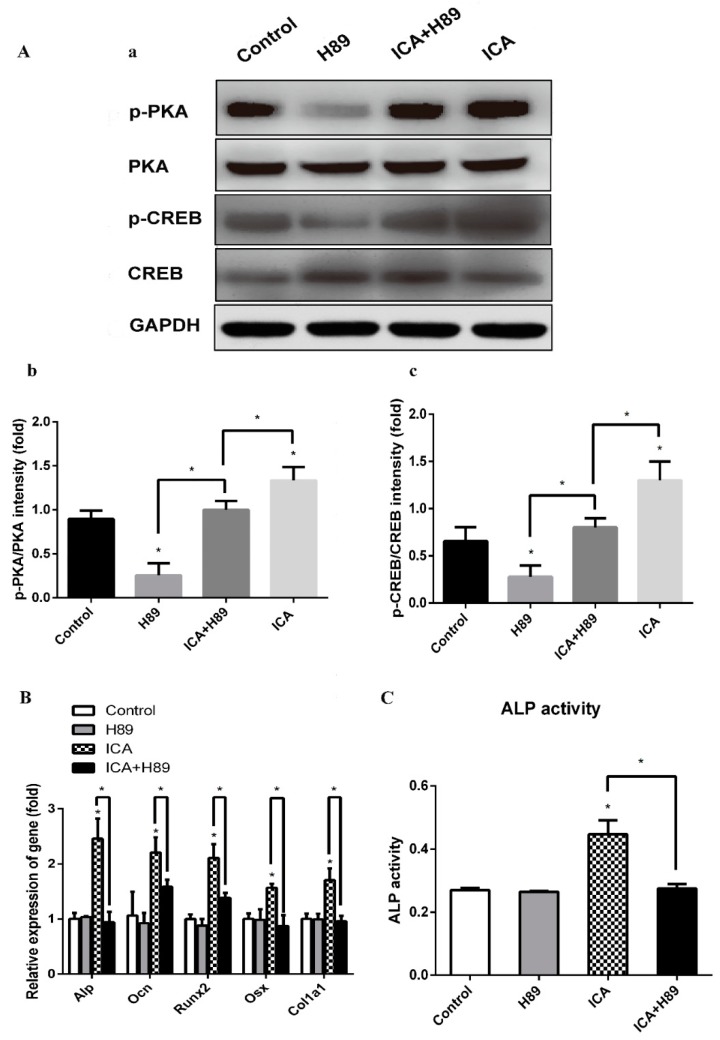
PKA inhibitor (H89) abrogated ICA-prompted osteogenesis via blockade of the cAMP/PKA/CREB signaling pathway. (**A-a**) The protein expression levels of p-PKA, p-CREB, total PKA and CREB treated with/without ICA and H89. (**A-b**) Relative density of p-PKA expression levels. (**A-c**) Relative density of p-CREB expression levels. The mRNA expression levels (**B**) and the ALP activity (**C**) on day 7 of osteogenic induction with/without ICA and H89. Bar represents the mean ± SD (*n* = 3). * *p* < 0.05.

**Figure 7:**
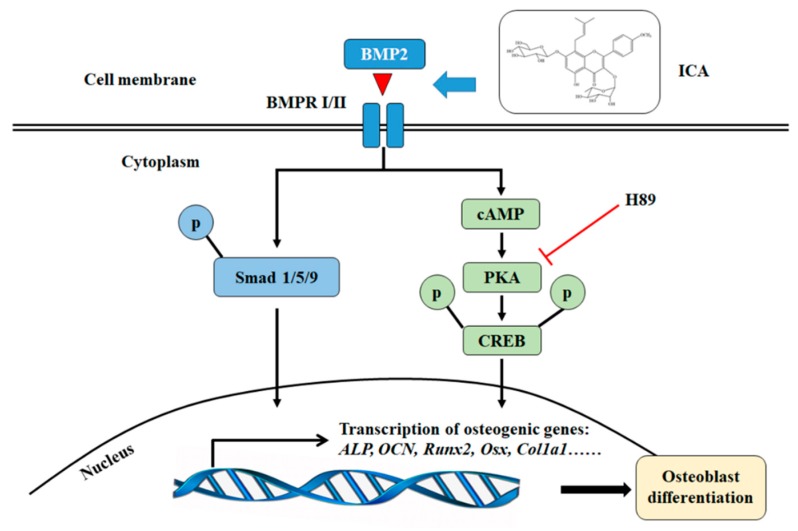
The schematic diagram of ICA activation of BMP2–induced osteoblast differentiation in C2C12 cells. ICA can stimulate the osteogenic activity of C2C12 cells with BMP-2 through the Smad1/5/9 and the cAMP/PKA/CREB signaling pathways.

To investigate the intracellular signaling pathway involved in the mechanism for the enhancement of osteogenic differentiation by ICA, we examined the effects of a PKA inhibitor (H89). C2C12 cells were pretreated with H89 (3 × 10^−5^ M) for 24 h followed by treatment with BMP2 (300 ng/mL) and ICA (10^−5^ M) and incubated for an additional 7 days. Then, we examined various osteogenic genes and the ALP activity. As shown in Figure 6A, the ICA-induced phosphorylation of PKA and CREB was significantly abrogated by H89, while there were no changes in the expression of total PKA and CREB, indicating that ICA-induced activation of the cAMP/PKA/CREB pathway was significantly blocked by H89. As shown in Figure 6B, the mRNA expression levels of *Alp*, *Ocn*, *Runx2*, *Osx* and *Col1a1* in the ICA group were significantly higher than those in the control group. There was no significant difference between the control group and the H89 alone group. However, the mRNA expression levels in the ICA+H89 group were dramatically lower than those in the ICA group, indicating that H89 abolished the stimulative action of ICA on various osteogenic differentiation markers almost completely. Similarly, as shown in Figure 6C, the ALP activity of the ICA group was significantly higher than that of the control group. There was no significant difference between the control group and the H89 alone group. However, the increase was abolished in the ICA+H89 group. All changes were similar to the changes in mRNA expression levels. Taken together, these results indicate that ICA could enhance the BMP2-mediated osteoblast differentiation of C2C12 cells by activating the cAMP/PKA/CREB signaling axis. Thus, as shown in Figure 7, ICA could promote the Smad1/5/9 and cAMP/PKA/CREB signaling pathways that led to cooperative activation of osteoblast differentiation.

## 3. Discussion

In summary, this study investigated the effects of ICA on BMP2-mediated osteogenic differentiation of C2C12 cells and the underlying molecular mechanisms. The simultaneous administration of BMP2 and ICA can strongly enhance the ALP activity and mRNA expression of osteogenic markers in a dose-dependent manner. This combination also activates the cAMP/PKA/CREB signaling axis, which is involved in the ICA-mediated promotion of the BMP2-induced osteoblastic differentiation of C2C12 cells. Blocking cAMP signaling using the PKA selective inhibitor H89 significantly abolished the stimulatory effect of ICA on osteogenesis. We concluded that the osteoinductive ability of ICA was mediated by the cAMP/PKA/CREB signaling pathway.

As is known, bone remodeling occurs continually and is mediated through the cycle of bone resorption and bone formation [26]. Bone remodeling is executed by two distinct types of cells: osteoblasts and osteoclasts. Osteoblasts promote bone formation via mineralization of collagen, while osteoclasts promote bone resorption by digesting bone minerals and proteins. When bone metabolism is disrupted, with bone resorption exceeding bone formation, osteoporosis will occur [27]. Osteoporosis is a bone disease that poses a significant risk to public health and is characterized by reduced bone mass. Long-term illness leads to an increased risk of bone fractures and causes a large social and economic burden. The traditional Chinese medicinal *Herba Epimedii* has been prescribed for the treatment of bone fractures and osteoporosis as a bone-protecting agent for centuries. ICA is a flavonoid glucoside extracted from herbs from the genus *Epimedium*, and ICA has been shown to be the main active component. Studies have shown that ICA exhibits potent estrogenic bioactivity and antihyperglycemic effects, which can protect rat models from osteoporosis induced by ovariectomy, glucocorticoids or diabetes [28,29,30,31,32]. Moreover, ICA can prevent osteoporosis in osteoprotegerin (OPG) KO mice, another novel animal model of osteoporosis, and this effect was mainly related to its ability to inhibit bone loss [33]. More recent studies have shown that ICA played a vital role in osteogenic differentiation in BMSCs and various other osteoblasts [34,35,36]. In addition to the osteogenic effects, ICA was shown to inhibit the formation of several osteoclasts and prevent bone loss in multiple studies [18,37,38]. Another research found that ICA dose-dependently inhibited the growth and differentiation of hemopoietic cells from which osteoclasts were formed [39]. ICA-mediated osteogenesis is the result of multiple signaling transduction pathways. Many studies have shown that ICA exerts its potent osteogenic effect by activating the BMP [36], NO [40], and Wnt signaling pathways [41], and downregulating the ERK and JNK pathways [42]. ICA acted on monocyte/macrophage-derived cells (pre-osteoclasts) or mature osteoclasts and inhibited their differentiation by regulating RANKL, OPG, ALP, and Col1 [37,43]. Meanwhile, another strategy to reduce bone loss was alleviation of inflammatory signaling pathways by ICA [18]. Recently, a study demonstrated that ICA promoted osteoblastic gene expression by activating the cAMP/PKA/CREB pathway [44]. Our experiments confirmed that ICA could enhance the osteogenic effect of BMP2 and that the cAMP-mediated signaling pathway activated by ICA was involved in BMP2-induced osteoblastic differentiation.

Many studies have suggested that the cAMP signaling pathway is required for regulating osteogenic differentiation and bone formation. Parathyroid hormone (PTH) is currently the only FDA-approved anabolic medicine for osteoporosis in the USA [45]. Many studies have indicated that the activation of the cAMP/PKA pathway, which is specifically stimulated by PTH-induced Gs coupling to PTH1R, is associated with PTH-induced osteogenic differentiation [19,20,46]. The role of the cAMP/PKA pathway in osteogenesis is further supported by several studies. Activation of the cAMP pathway in primary aortic medial cells promoted mineralization and vascular calcification by enhancing osteoblast-like differentiation [47]. cAMP/PKA activation resulted in robust osteogenic differentiation in vivo by transplantation after pretreatment of human MSCs via the cAMP analog or forskolin [21,22]. Pentoxifylline, a nonselective phosphodiesterase (PDE) inhibitor, can promote osteoblastic differentiation by elevating the intracellular cAMP levels and enhancing bone formation in vivo and in vitro [48]. Another study indicated that the anabolic effect of cAMP on BMP2-induced osteoblastic differentiation is mediated by enhancing the transcriptional activity of the BMP response element (BRE) through the cAMP response element (CRE), in which the PKA signaling pathway is involved [49]. This conclusion is also consistent with our results, in which the ICA-enhanced elevation of BMP2-induced osteoblastic differentiation in C2C12 cells was significantly decreased by H89 treatment. These observations strongly suggest that cAMP/PKA is involved in the enhancement of BMP2-induced transcriptional activity by ICA. 

However, this study has some limitations. First, the exact molecular mechanism of how the activated cAMP/PKA/CREB signaling pathway enhances BMP2-mediated osteogenic differentiation was not determined. Second, in-depth in vivo studies are needed to confirm these hypotheses. Further work is underway in our laboratory to achieve these goals.

These observations add to our knowledge of the osteogenic effects of ICA and form a molecular basis for the development of bone anabolic drugs. From a clinical perspective, the ICA used in our study could be a promising agent for osteogenic differentiation because, compared to BMP2 alone, the addition of ICA dramatically enhanced the performance of BMP2 and increased bone formation.

## 4. Materials and Methods 

### 4.1. Reagents 

ICA reagents (purity > 99%) were purchased from the National Institute for the Control of Pharmaceutical and Biological Products (Beijing, China) and dissolved in DMSO. H89 was purchased from Sigma-Aldrich (St. Louis, MO, USA). BMP2 was purchased from PeproTech (Rocky Hill, NJ, USA). Antibodies against phosphorylated Smad1/5/9, Smad1, phosphorylated CREB and CREB were obtained from Cell Signaling Technology (Danvers, MA, USA). Antibodies against phosphorylated PKA and PKA were purchased from Abcam (Cambridge, UK). Cellular nuclear DNA was stained with DAPI, which was obtained from Sigma (St. Louis, MO, USA). 

### 4.2. Cell Culture 

Mouse myoblast C2C12 cells were purchased from American Type Culture Collection (ATCC) and cultured in Dulbecco’s modified Eagle’s medium (DMEM) with 10% fetal bovine serum (FBS; Gibco, CA, USA) and 1% penicillin/streptomycin in 5% CO_2_ at 37 °C. Cells were seeded; on the second day, the cells were differentiated by replacing the medium with differentiation medium (DMEM containing 5% FBS and 300 ng/mL BMP2). The BMP2 medium group was defined as the control group. ICA was complementally added at 10^−8^, 10^−7^, 10^−6^, 10^−5^ and 10^−4^ M. The concentration of DMSO (as the solvent of ICA) in culture was restricted to 0.05%, a safe concentration that did not harm the cells [50]. The culture media were replaced every 2 days.

### 4.3. Cytotoxicity Assay 

To investigate the cytotoxicity of ICA, C2C12 cells were plated on 96-well plates (5,000 cells/well). After preincubation for 24 h in a humidified incubator, fresh media containing different concentrations of ICA were added. The cell numbers at 0, 1, 2, 3, 5 and 7 days were used as indicators of cell viability and determined using a Cell Counting Kit-8 (Dojindo, Kumamoto, Japan) according to the manufacturer’s protocol. The optical density (OD) values were measured at 450 nm. 

### 4.4. Alkaline Phosphatase (ALP) Activity Assay

C2C12 cells were seeded into 24-well plates at a density of 2 × 10^4^ cells per well and incubated in a humidified atmosphere of 37 °C and 5% CO_2_. At confluence, cells were cultured in BMP2 with ICA for 1, 3, 5 and 7 days. At the end of culturing, cells were gently washed twice with PBS and then lysed with 0.2% Triton X-100, and the lysate was centrifuged at 14,000 g for 15 min. The supernatant was collected for the measurement of ALP activity by an ALP activity assay kit (Nanjing Jiancheng Bioengineering, Ltd., Nanjing, China), and the protein concentrations were determined by a BCA-protein assay kit (Beyotime Institute of Biotechnology, shanghai, China).

### 4.5. Transcriptome Sequencing 

The gene expression profile of ICA-induced osteoblast differentiation was analyzed with RNA-seq technology. When cultures in 25 cm^2^ culture bottles reached confluence, the medium was replaced by fresh medium with ICA and/or BMP2 (BMP2 alone medium served as the control group), and incubation was continued for 7 days. First, total RNA was extracted using QIAamp RNA Blood Mini Handbook (QIAGEN, Germantown, MD, USA). Thereafter, the quality and quantity of RNA were assessed using a NanoPhotometer ^®^ spectrophotometer (IMPLEN, Los Angeles, CA, USA) and an Agilent 2100 Bioanalyzer (Agilent Technologies, Palo Alto, CA, USA). Sequencing libraries were generated using VAHTSTM mRNA-seq V2 Library Prep Kit for Illumina^®^ following the manufacturer’s recommendations, and index codes were added to attribute sequences to each sample. Briefly, mRNA was purified from total RNA using poly T oligo-attached magnetic beads. To preferentially select cDNA fragments that were 150~200 bp in length, the library fragments were purified with AMPure XP system (Beckman Coulter, Beverly, CA, USA). At last, PCR products were purified (AMPure XP system) and library quality was assessed on the Agilent Bioanalyzer 2100 system. The libraries were then quantified and pooled. After RNA-seq library construction, paired-end sequencing of the library was performed on HiSeq XTen sequencers (Illumina, San Diego, CA, USA). DESeq2 (version 1.12.4) was used to determine differentially expressed genes (DEGs) between two samples. Genes were considered to be significantly differentially expressed if the q-value < 0.001 and |FoldChange| >2. Gene Ontology (GO) and Kyoto Encyclopedia of Genes and Genomes (KEGG) were performed to identify which DEGs were significantly enriched in GO terms or metabolic pathways.

### 4.6. cAMP Assay 

When cultures in 24-well plates reached confluence, the medium was replaced by fresh medium with BMP2 and ICA, and incubation was continued for 0, 15, 30, 45, 60 and 120 min. Then, we removed the medium and determined the amounts of cAMP in wells with a cAMP enzyme immunoassay system (R&D Systems, Minneapolis, MN, USA) according to the manufacturer’s instructions. 

### 4.7. qRT-PCR Analysis 

C2C12 cells were seeded into 6-well plates and treated with BMP2 (300 ng/mL or absent), ICA (10^−6^, 10^−5^ M or absent) and H89 (3 × 10^−5^ M or absent) for 1, 3, 5 and 7 days. Total RNA was extracted using TRIzol Reagent (Invitrogen, Albuquerque, NM, USA) according to the manual. Then, the RNA samples were reverse transcribed into cDNA using a PrimeScript RT Reagent Kit with gDNA Eraser (TaKaRa, Japan). Real-time quantitative PCR detection was performed with FastStart Universal SYBR Green Master (Rox) on a LightCycler^®^ 480 II Real-Time PCR System (Roche, Mannheim, Germany). All reactions were run in triplicate, and data were analyzed using the 2^−ΔΔCt^ method normalized to GAPDH. The primer sequences are listed in Table 1. 

### 4.8. Western Blot Analysis 

Cells seeded in 25 cm^2^ culture bottles were lysed with cold RIPA Lysate reagent (Beyotime Institute of Biotechnology, Shanghai, China) according to the manufacturer’s instructions. The protein concentrations were measured with the BCA assay kit. Proteins (40 µg) were separated by 12% SDS-PAGE and transferred to 0.45 µm PVDF membranes (Merck Millipore, Darmstadt, Hesse-Darmstadt, Germany). After the membranes were blocked, they were incubated overnight at 4 °C with the diluted primary antibodies against the following molecules: phosphorylated Smad1/5/9, Smad1, phosphorylated PKA, PKA, phosphorylated CREB, CREB and GAPDH. Then, the membranes were washed with TBST and incubated with diluted secondary antibodies for 1 h at room temperature. Finally, we visualized the membranes with an enhanced ECL substrate kit (Millipore, Billerica, MA, USA) on the Fusion SOLO S (Vilber, Collégien, France). The densities of the product bands were quantified using ImageJ software and standardized to that of GAPDH.

### 4.9. Immunofluorescence Staining 

Cells that migrated to the coverslip in 24-well plates were fixed with 4% paraformaldehyde for 30 min and washed three times with PBS. Then, the cells were permeabilized with 0.5% Triton X-100 for 20 min. Subsequently, the cells were blocked in 5% goat serum for 1 h at room temperature and then incubated with an anti-phosphorylated PKA antibody overnight at 4 °C. The next day, we rewarmed the cells at room temperature for 1 h and then incubated the cells with a goat anti-rabbit conjugated secondary antibody for 1 h at 37 °C in the dark. Finally, the cells were incubated with DAPI for 5 min. The slides were examined using a 3D scanner (3D HISTECH, Budapest, Hungary).

### 4.10. Statistical Analysis 

All data are expressed as the mean ± SD. Each treatment group had at least three replicates (n = 3), and each experiment was repeated 3 times. Statistical analyses were carried out with PRISM (version 6, GraphPad Software, Inc., San Diego, CA, USA). In all cases, *p* < 0.05 was considered significant.

## Figures and Tables

**Table 1:** Primers used for qRT-PCR.

Name	Sequence
*Alp*	3′→5′: GGCTCTGCCTTTATTCCCTAGT 5′→3′: AAATAAGGTGCTTTGGGAATCTGT
*Ocn*	3′→5′: GCCATCACCCTGTCTCCTAA 5′→3′: GCTGTGGAGAAGACACACGA
*Runx2*	3′→5′: GCCGGGAATGATGAGAACTA 5′→3′: GGTGAAACTCTTGCCTCGTC
*Osx*	3′→5′: AGGCCTTTGCCAGTGCCTA 5′→3′:GCCAGATGGAAGCTGTGAAGA
*Col1a1*	3′→5′: GACATGTTCAGCTTTGTGGACCTC 5′→3′:GGGACCCTTAGGCCATTGTGTA
*Gapdh*	3′→5′: CATCCCAGAGCTGAACG 5′→3′: CTGGTCCTCAGTGTAGCC
